# General Error Analysis in the Relationship between Free Thyroxine and Thyrotropin and Its Clinical Relevance

**DOI:** 10.1155/2013/831275

**Published:** 2013-09-08

**Authors:** Simon L. Goede, Melvin Khee-Shing Leow

**Affiliations:** ^1^Electronic Engineering, Oterlekerweg 4, 1841 GP Stompetoren, The Netherlands; ^2^Department of Endocrinology, Tan Tock Seng Hospital, 11 Jalan Tan Tock Seng, Singapore 308433; ^3^Singapore Institute for Clinical Sciences, Brenner Centre for Molecular Medicine, 30 Medical Drive, Singapore 117609; ^4^Yong Loo Lin School of Medicine, Centre for Translational Medicine, National University of Singapore, 14 Medical Drive, No. 07-02, Singapore 117599; ^5^Office of Clinical Sciences, Duke-NUS Graduate Medical School, 8 College Road, Singapore 169857

## Abstract

*Background*. This treatise investigates error sources in measurements applicable to the hypothalamus-pituitary-thyroid (HPT) system of analysis for homeostatic set point computation. The hypothalamus-pituitary transfer characteristic (HP curve) describes the relationship between plasma free thyroxine [FT4] and thyrotropin [TSH]. *Objective*. We define the origin, types, causes, and effects of errors that are commonly encountered in TFT measurements and examine how we can interpret these to construct a reliable HP function for set point establishment. *Design and Methods*. The error sources in the clinical measurement procedures are identified and analyzed in relation to the constructed HP model. *Results*. The main sources of measurement and interpretation uncertainties are (1) diurnal variations in [TSH], (2) TFT measurement variations influenced by timing of thyroid medications, (3) error sensitivity in ranges of [TSH] and [FT4] (laboratory assay dependent), (4) rounding/truncation of decimals in [FT4] which in turn amplify curve fitting errors in the [TSH] domain in the lower [FT4] range, (5) memory effects (rate-independent hysteresis effect). *Conclusions*. When the main uncertainties in thyroid function tests (TFT) are identified and analyzed, we can find the most acceptable model space with which we can construct the best HP function and the related set point area.

## 1. Introduction

The precision of routine automated immunoassays such as thyroid function tests (TFT) is generally assumed unquestionably reliable and robust for clinical decision making with respect to diagnosis and treatment. However, we have come to realize that there is a fundamental difference between measurement accuracy and measurement sensitivity.

Recent overviews of the sensitivity, accuracy, and quality of modern immunoassays [1, 2] give a more laboratory-oriented and technical insight into the possibilities of various test kits. Most test kits obtain a measurement inaccuracy of about 5% both for [TSH] and [FT4]. The measurement sensitivity is a different matter. Here we see the improvement of detection level technology. For the detection of [FT4], currently, the sensitivity can obtain measurement results with one decimal. [TSH] detection is even more sensitive where the measured values of [TSH] can be presented with two decimals. The sensitivity has increased over the years, and the accuracy of the reference standards for these measurements is generally limited to 5%. This accuracy figure will also be used as a lead in the general discussion about this topic. The assay reference accuracy and the sensitivity of detectable [TSH] and [FT4] concentrations are subject to a closer inspection and investigation related to the practical interpretation of these measurements.

This treatise highlights, examines, and interprets the nature and sources of common errors that are encountered in measurements and interpretation of the presented data of plasma free thyroxine [FT4] and thyrotropin [TSH] and their impact on thyroid modeling research and clinical practice, hitherto underestimated. 

General clinical research is mostly related to a trial and examination of a certain population using the statistical computation of a large aggregate of measured data which are interpreted as the resulting mean values, regression analysis, and trends based on the given measurements. Such a method will not result in any specifics about an individual. This is possibly the reason why some uncertainties and measurement inaccuracies will not be a major factor when most variability will be cancelled out in a large population. Any modeling attempt in this respect will provide a result that is too generalized to be of any use in an individual application especially with respect to elucidating the physiological and metabolic responses of living organisms including humans. 

In this treatise, our area of interest is in the accurate modeling of the hypothalamus-pituitary-thyroid (HPT) axis, whereby the hypothalamus and pituitary components are mathematically integrated for the sake of simplification into a combined hypothalamic-pituitary (HP) unit. We want to construct a specific individual HP characteristic based on personalized measurements where the TFT errors and interpretation of the measurements can have a considerable impact on the quality of the obtained HP model result.

This is particularly relevant in this modern era of personalized medicine where inaccuracies of [FT4] and [TSH] can compromise individualization of patient-specific treatment endpoints based on mathematical models for homeostatic set point determination. The results of a formal theoretical analysis of these errors and interpretation of relevant deviations will enable clinicians to better understand how to minimize these effects particularly when applying algorithms for individualizing set points to achieve precise and optimal treatment for their patients. 

The current literature reveals that a negative exponential model fits clinical data best with respect to the relationship between [TSH] and [FT4] and is thus far the most tenable and enduring of all existing theoretical constructs [3, 4]. Although the formal derivation from physiological data [3] reveals the exponential relationship, the validation is verified on an individual set of clinical TFTs.

Former existing publications using scatter plots of homeostatic values of hundreds of measured [TSH]-[FT4] combinations from different individuals [5, 6] hinted at a “logarithmic [TSH]-linear [FT4]” relationship based on regression analyses. Scatter plots are two-dimensional representations of a certain distribution of [TSH], coupled with a belonging [FT4] value. It is helpful to remember that the normal reference ranges of [TSH] and [FT4] are established by each clinical laboratory via the statistical distribution of [TSH] and [FT4] of a group of healthy subjects from the general population. In these scatter plots, every coupled [TSH]-[FT4] coordinate represents the current value of the points of equilibrium of individual thyroid status in the investigated population. Since the physiological processes and parameters of each individual are unique as a fingerprint and different people can thus have widely disparate [TSH]-[FT4] pairs on such plots despite having similar thyroid hormone status, it is impossible from these published scatter plots and their related regression analyses to draw any valid conclusion about the individual [TSH]-[FT4] relationship within any given person across the spectrum of thyroid hormone status.

However, the scatter plots have additional qualities which can provide an unexpected insight into modeling. For instance, there are persons with nearly similar euthyroid set points despite different HP physiological processes and parameters. There are also people with distinctive deviations of the normal set point albeit with relatively similar HP characteristics. The HP characteristic curve should therefore be considered as the geometrical collection of all possible points of equilibrium of an individual, including the set point. 

Variations of euthyroid TFTs of any given individual are distributed over such a narrow margin that we can assume a stable set point position over a relatively long period of time [7]. It can be readily appreciated that some individuals have a naturally “high normal” [FT4] with an accompanying “low normal” [TSH], whereas others are euthyroid with a “low normal” [FT4] and relatively “high normal” [TSH]. In all cases, we find a distinct negative exponential relationship. The formal derivation of the [TSH]-[FT4] relationship as shown in [4] provides the insight into the individual modeling with two independent model parameters. These individual parameters can be calculated from the revenant series of personal TFTs. The proposed parameterized negative exponential model derived from earlier published results [4] here is

(1) [TSH]=Sexp⁡(−φ[FT4]).

Based on recent unpublished validation of the individual HP function, we postulate this characteristic as a two-dimensional (*S* and *φ*), parameterizable negative exponential function that forms the basis for the interpretation of [TSH] and related [FT4] measurements.

The HP characteristic has by the intrinsic nature of exponential functions the following properties that should be elaborated on.

(1) One and only one exponential function is completely defined by the coordinates of two different points according to
  (2) $$\begin{array}{c} \varphi =\left(\frac{1}{\left({\left[\text{FT}4\right]}_{1}-{\left[\text{FT}4\right]}_{2}\right)}\right)\ln\left(\frac{{\left[\text{TSH}\right]}_{2}}{{\left[\text{TSH}\right]}_{1}}\right), \end{array}$$
  (3) $$\begin{array}{c} S={\left[\text{TSH}\right]}_{1}\exp\left(\varphi {\left[\text{FT}4\right]}_{1}\right)={\left[\text{TSH}\right]}_{2}\exp\left(\varphi {\left[\text{FT}4\right]}_{2}\right), \end{array}$$
  (4) $$\begin{array}{c} P_{1}=\left({\left[\text{FT}4\right]}_{1},{\left[\text{TSH}\right]}_{1}\right),\;\;P_{2}=\left({\left[\text{FT}4\right]}_{2},{\left[\text{TSH}\right]}_{2}\right). \end{array}$$
(2) There is a unique point on an exponential function, the point of maximum curvature, from which the complete function can be reconstructed.
 In that case, the function will be completely determined by one point.

This point of maximum curvature and thus maximal sensitivity for change can be found according to the standard curvature theory. 

The radius of curvature at a point of a function is defined as *R* and the curvature is defined as *K*. Thus,

(5) $$\begin{array}{c} K=\frac{1}{R}. \end{array}$$

If the function to be examined is defined as *y* = *f*(*x*), then the curvature *K* of *y* is defined as

(6) $$\begin{array}{c} K=\frac{f^{\prime \prime }\left(x\right)}{{\left(1+{\left(f^{\prime }\left(x\right)\right)}^{2}\right)}^{3/2}}. \end{array}$$

With these fundamental function properties and the fact that the HP characteristic is the individual geometrical collection of all possible homeostatic positions, we discuss the interpretation of the [FT4]-[TSH] measurements. 

The postulated model of (1) is the integral response of all underlying physiological processes related to the regulation of the thyroid hormone system. Furthermore, it should be noted that the response of this hormone system to homeostatic results has a time constant of about four to six weeks.

This model has two degrees of freedom, *S* and *φ*. These degrees of freedom open the possibility to position the graph of the model anywhere on a two-dimensional Euclidean plane. The model can be validated with measurements of [FT4]-[TSH] pairs of persons with overt primary hypothyroidism treated with L-T4 over a number of months till their [FT4] and [TSH] are fine-tuned to the neighborhood of their original predisease euthyroid set point. 

Several validation examples (unpublished data) from different laboratory assays are available showing the general validity of the model. It should be noted that every individual has his or her own distinct personal characteristic HP curve. This uniqueness is shown by individually calculated values for *S* and *φ*. The goodness of fit (*R* ^2^) of the model was in general better than 95%. The validation and construction processes can only be done correctly when certain conditions of the performed measurements are met and interpreted accordingly. As previously mentioned, it should be noted that the HP curve is theoretically defined by a minimum of two measured sets of [FT4] and [TSH] as illustrated by the formulations (2), (3), and (4).

## 2. Measurement Qualifiers and Sensitivity Analysis

### 2.1. Diurnal Variations in [TSH]

Biological variations are expected to occur in the [FT4] and [TSH]. A very important condition for TFT measurements is the time of day the blood is sampled as many hormone systems in the body exhibit natural circadian biorhythms dependent on time including the HPT axis. The diurnal rhythm of [TSH] plays an important role and exhibits a significant difference between morning and evening readings [8]. The reported variations in [TSH] levels are ranging from an average [TSH] of 1 mU/L at about 15.00 h in the afternoon to an average of [TSH] = 2 mU/L at about midnight. This reveals the importance of a repeatable defined measurement regime at a fixed time of day. Any measurement accuracy of [TSH] to the extent we have available loses significance if we ignore these effects.

The amount of variability is dependent on the individual in question, but the smallest interindividual variations in [TSH] are observed around 15.00 h in the afternoon [8].

In another study [9], it was evident that several persons being probed for FT4 after taking their daily dose of levothyroxine (L-T4) had different readings because of interindividual differences in pharmacokinetics and metabolism. Therefore it is important to probe a person already using L-T4 on a fixed time of the day before the intake of the daily L-T4 dose. For practical reasons, this can be done shortly upon awakening (i.e., prior to the ingestion of daily dose of L-T4) in the early morning between 07.00 h and 10.00 h. The same time interval for TFT assessment also applies to people being investigated for the first time. 

### 2.2. Presentation Inaccuracies from [FT4] Rounding or Truncation Procedure

The following discusses the effects of approximation by “rounding” and “truncation” of decimals of measured [FT4] values, provided by the clinical laboratory, in an [FT4]-[TSH] plot. [FT4] measurements can currently be carried out with a relative accuracy of at least 5% [2]. In many clinical practices, the presentation on [FT4] is given in integers. This implies an uncertainty of the real value measured. When only rounding to the nearest integer is provided, we can expect an absolute uncertainty of ±0.5 pmol/L. Sometimes, the [FT4] results are presented as truncated decimal readings. Let us consider the following instances in which an [FT4] value of 12.7 pmol/L is presented in truncated form as 12 pmol/L and another measured [FT4] result of 13.2 pmol/L is reported as 13 pmol/L. This procedure implies an uncertainty in the [FT4] results as ±1 pmol/L. 

**Figure 1:**
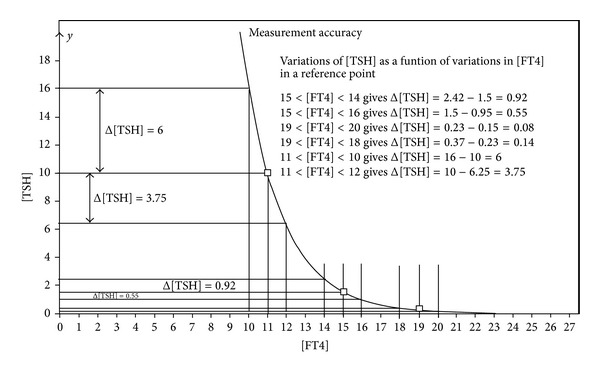
[TSH] sensitivity for variations in [FT4].

Although many clinical and biochemical laboratories in Singapore follow a practice of expressing [FT4] to the first decimal place, there are also laboratories which either round off the value of [FT4] to the nearest integer or truncate away decimals and leave behind only the integer. This is basically a practice to avoid misleading clinicians to overinterpret trivial changes in serial results which may actually reflect analytical and biological “noises”. Equally, there are also laboratories which are similarly erroneous by using the default number of decimal places that appear on the analyzer printout without making adjustments to match the analytical imprecision of their assays. In most Dutch laboratories, the rounding of the [FT4] results in one significant decimal place value digit (tenth). For example, [FT4] = 12.7 pmol/L would be a typical reported value. In Figure 1, we have an example of the HP characteristic presented as a median representation.

Furthermore, it shows the effects in a certain part of the HP characteristic when the rounding procedure is performed on one single [FT4] measurement resulting in a rounded integer. This means that the maximum rounding error can be defined as [FT4] ±0.5 pmol/L. The consequences for the error magnitude depend strongly on the position on the curve. For example, a measured value of [FT4] = 11.4 pmol/L will be rounded down to 11 pmol/L, while a measured value of 11.6 pmol/L will be rounded to up to 12 pmol/L. In the first case, we will find an error in [TSH] of 6 mU/L. In the second instance, the error will be 3.75 mU/L. This rounding error will decrease for the higher ranges of [FT4]. In the present discussion, it is important that the predicted error magnitudes in [TSH] apply insofar as the negative exponential model fitting is concerned and not to be confused with actual coefficient of variations (CV) of the [TSH] assay. 

### 2.3. Absolute Deviation of [FT4] as a Function of Relative [TSH] Error

Using the dependency expression of [FT4] and [TSH],

(7) $$\begin{array}{c} \left[\text{TSH}\right]=\frac{S}{\exp\left(\varphi \left[\text{FT}4\right]\right)}. \end{array}$$

We will investigate the deviation effects on [FT4] as a function of the relative error in [TSH]. Suppose the relative error in [TSH] is represented by *p*%; then the upper limit value of [TSH] is

(8) $$\begin{array}{c} \text{U}{\text{L}}_{\left[\text{TSH}\right]}=\left[\text{TSH}\right]\left(1+\frac{p}{100}\right), \end{array}$$

and the lower limit will be

(9) $$\begin{array}{c} \text{L}{\text{L}}_{\left[\text{TSH}\right]}=\left[\text{TSH}\right]\left(1-\frac{p}{100}\right). \end{array}$$

This has the following effect on the related values for [FT4].

From (3), we can write [FT4] as

(10) $$\begin{array}{c} \left[\text{FT}4\right]=\frac{1}{\varphi }\ln\left(\frac{S}{\left[\text{TSH}\right]}\right). \end{array}$$

The absolute difference in the related values for [FT4] as the upper limit of [FT4] is

(11) $$\begin{array}{c} \text{U}{\text{L}}_{\left[\text{FT}4\right]}=\frac{1}{\varphi }\ln\left(\frac{S}{\text{L}{\text{L}}_{\left[\text{TSH}\right]}}\right), \end{array}$$

and the lower limit of [FT4] is

(12) $$\begin{array}{c} \text{L}{\text{L}}_{\left[\text{FT}4\right]}=\frac{1}{\varphi }\ln\left(\frac{S}{\text{U}{\text{L}}_{\left[\text{TSH}\right]}}\right). \end{array}$$

The absolute difference between the values of [FT4] is then expressed as

(13) $$\begin{array}{c} \text{U}{\text{L}}_{\left[\text{FT}4\right]}-\text{L}{\text{L}}_{\left[\text{FT}4\right]}=\frac{1}{\varphi }\ln\left(\frac{S}{\text{L}{\text{L}}_{\left[\text{TSH}\right]}}\right)-\frac{1}{\varphi }\ln\left(\frac{S}{\text{U}{\text{L}}_{\left[\text{TSH}\right]}}\right),\\ \text{U}{\text{L}}_{\left[\text{FT}4\right]}-\text{L}{\text{L}}_{\left[\text{FT}4\right]}=\frac{1}{\varphi }\left(\ln\left(\frac{S}{\text{L}{\text{L}}_{\left[\text{TSH}\right]}}\right)-\ln\left(\frac{S}{\text{U}{\text{L}}_{\left[\text{TSH}\right]}}\right)\right),\\ \text{U}{\text{L}}_{\left[\text{FT}4\right]}-\text{L}{\text{L}}_{\left[\text{FT}4\right]}=\frac{1}{\varphi }\left(\ln\left(\frac{\text{U}{\text{L}}_{\left[\text{TSH}\right]}}{\text{L}{\text{L}}_{\left[\text{TSH}\right]}}\right)\right). \end{array}$$

Substituting (4) and (5) into this expression, we find

(14) UL[FT4]−LL[FT4]=1φ(ln⁡([TSH](1+p/100)[TSH](1−p/100)))=1φ(ln⁡((1+p/100)(1−p/100)))

or

(15) $$\begin{array}{c} \Delta \left[\text{FT}4\right]=\frac{1}{\varphi }\left(\ln\left(\frac{\left(1+p/100\right)}{\left(1-p/100\right)}\right)\right). \end{array}$$

This means that the absolute error of [FT4] is a function of the relative error, *p*, of [TSH] and *φ* and is independent of *S* and is also constant over the entire range of [FT4]. 

This is a direct consequence of the exponential properties of the relationship between [FT4] and [TSH]. Another way of expressing the deviation in the value of [FT4] is

(16) $$\begin{array}{c} \left[\text{FT}4\right]=\pm 0.5\Delta \left[\text{FT}4\right]. \end{array}$$

The results presented in Table 1 illustrate the minimal impact of relative deviations of [TSH] with *p* = 1%, the most practical situation, on the absolute differences in [FT4].

At relative [TSH] errors of *p* = 5%, the influence on deviations in [FT4] is more obvious. The strongest deviation from this table is found when *p* of [TSH] = 5% and *φ* = 0.3, and then the absolute error of [FT4] is ±0.5Δ[FT4] = ±0.165 pmol/L.

This error is the same over the entire [FT4] reference range.

### 2.4. The Effect of Absolute Errors in FT4 Resulting in Deviations in [TSH]

The absolute error of plus or minus *λ* in the values of [FT4] can be expressed as

(17) $$\begin{array}{c} F_{\left([\text{FT}4]+\lambda \right)}=\frac{S}{\exp\left\{\varphi \left(\left[\text{FT}4\right]+\lambda \right)\right\}},\\ F_{\left([\text{FT}4]-\lambda \right)}=\frac{S}{\exp\left\{\varphi \left(\left[\text{FT}4\right]-\lambda \right)\right\}}. \end{array}$$

The resulting absolute error in the value of [TSH] can be written as

(18) $$\begin{array}{c} F_{\varepsilon }=F_{\left([\text{FT}4]-\lambda \right)}-F_{\left([\text{FT}4]+\lambda \right)}, \end{array}$$

resulting in

(19) $$\begin{array}{c} F_{\varepsilon }=\frac{S}{\exp\left\{\varphi \left(\left[\text{FT}4\right]-\lambda \right)\right\}}-\frac{S}{\exp\left\{\varphi \left(\left[\text{FT}4\right]+\lambda \right)\right\}}, \end{array}$$

resulting in

(20) $$\begin{array}{c} F_{\varepsilon }=\frac{S\left(\exp\left\{\varphi \left(\left[\text{FT}4\right]+\lambda \right)\right\}-\exp\left\{\varphi \left(\left[\text{FT}4\right]-\lambda \right)\right\}\right)}{\exp\left\{2\varphi \left[\text{FT}4\right]\right\}}. \end{array}$$

Thus

(21) $$\begin{array}{c} F_{\varepsilon }=\frac{S\exp\left\{\varphi \left[\text{FT}4\right]\right\}\left\{\exp\left(\varphi \lambda \right)-\exp\left(-\varphi \lambda \right)\right\}}{\exp\left\{2\varphi \left[\text{FT}4\right]\right\}}, \end{array}$$

and then

(22) $$\begin{array}{c} F_{\varepsilon }=\frac{S\left\{\exp\left(\varphi \lambda \right)-\exp\left(-\varphi \lambda \right)\right\}}{\exp\left\{\varphi \left[\text{FT}4\right]\right\}} \end{array}$$

or

(23) $$\begin{array}{c} F_{\varepsilon }=\frac{2S\sinh\left(\varphi \lambda \right)}{\exp\left\{\varphi \left[\text{FT}4\right]\right\}}. \end{array}$$

The effects of “*λ*” are shown as [FT4] ±0.5 compared to the results when *λ* = 0.1 as can be appreciated from Table 2.

The reduction in deviations of [TSH] is quite obvious when *λ* is reduced from 0.5 to 0.1. For a more accurate measurement result of [TSH] as a function of [FT4], it is recommendable to have [FT4] rounded off to the first decimal place instead of a whole integer. While this recommendation is elegantly justified mathematically in Table 2, it is also important for laboratories to be cognizant of the level of accuracy of their FT4 immunoassays platforms so that their chosen number of significant figures in their [FT4] reports is a realistic reflection of the assay system without overstating the number of decimal places beyond its performance characteristics.

### 2.5. Interpretation Errors from Physiological Memory Effects or Hysteresis

From clinical experience, we learned that healthy set points are expected in a certain area of the reference ranges of [FT4] and [TSH]. However, in cases of severe hyper- or hypothyroid conditions, the disordered physiology needs a significant amount of time to restore the system to normal system operation [4].

This “memory effect” in which a system is dependent not only on its current state but also on its former environment is recognized as hysteresis and is a phenomenon well characterized in many areas of physics such as what has been observed and analyzed in the magnetization curves of ferromagnetic materials. In the realm of physiology and medicine, hysteresis has also been encountered in pulmonary mechanics, bladder smooth muscle stretch, and even calcium-parathyroid hormone relationship [10–12].

With thyroid physiology, hysteresis causes a shift of the original characteristic over the horizontal axis, in this case the [FT4] axis. When the HPT axis encounters sustained elevated concentrations of [FT4] well above the extreme of the upper normal limit (i.e., thyrotoxicosis), it is consistently observed that the recovery response of [TSH] lags behind that of [FT4] with definitive treatment of the thyrotoxicosis before [TSH] will be detectable again even when [FT4] declines to subnormal levels. Evidently, the system “remembers” the huge amount of [FT4] and needs time to remove the effects. This is actually a beneficial adaptive feature that is evolutionarily conserved to protect the organism in case [FT4] should suddenly escalate rapidly without restraint, since the [TSH] will remain low and will not add undue further stimulation to the thyroid. When the [TSH] response is detectable again, we see a shift of the original HP characteristic to the lower end of the range of the [FT4] scale. 

A similar effect is observable after a long standing hypothyroid condition with [FT4] concentrations depressed far below the lower normal limit. In this case, the [TSH] normalization response lags behind the recovery of [FT4] such that [TSH] may still remain in the supranormal levels despite [FT4] having achieved normal or even relatively high levels due to the HP curve being shifted to the higher range of the [FT4] values. Again, this hysteresis represents a protective mechanism as the persistently elevated [TSH] provides a “buffer” of additional thyroid stimulation in case [FT4] should suddenly diminish to negligible from an unpredictable loss of exogenous T4 supply.

### 2.6. Hysteresis Modeling

We can remodel the original HP function and generalize it to an extended one where the saturation effects can be taken into account. The earlier form of the HP characteristic is still present. However, at lower values of [FT4], the saturation effect will be dominant.

The extended model can be written as

(24) $$\begin{array}{c} \left[\text{TSH}\right]=\frac{S}{\alpha S+\exp\left(\varphi \left[\text{FT}4\right]\right)}. \end{array}$$

**Figure 2:**
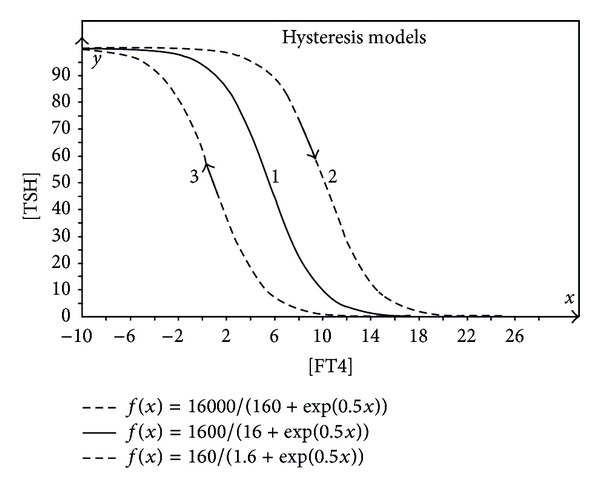
Expanded view of the hysteresis effect.

Here, *S* has the same meaning as in the original HP function, as the same also holds true for the value of *φ*. The new model parameter *α* introduces a new degree of freedom defining the boundary of the maximum [TSH] secretory capacity of the pituitarygland. In the current example, the normalized value of *α* = 1/100 results in a maximum pituitary secretory capacity corresponding to a [TSH] concentration of 100 mU/L. The hysteresis effect is in essence a nonlinear memory effect and can practically be described by the same function of which only the value of *S* will change as is shown in Figure 2.

In Figure 2, the hysteresis effect takes place starting in the normal (solid) “curve 1” with decreasing [FT4] where [TSH] is reaching the area of saturation ([TSH] = 100 mU/L).

**Figure 3:**
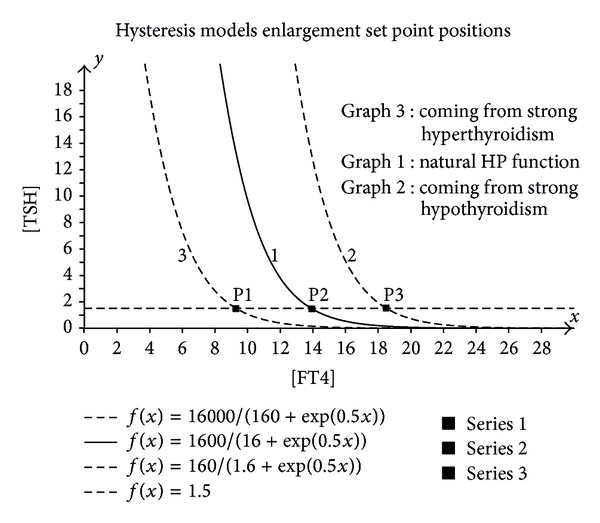
Enlargement of the set point positions.

The saturation results in a physiological condition such that it will take a certain amount of increased [FT4] after which the saturation effect has been compensated resulting in dotted “curve 2” and all physiological processes and conditions have been returned to the nonsaturated situation. The normal physiological response can take place with a shifted HP characteristic “curve 2” and therefore a shifted set point position as shown in Figure 3.

When coming from a very high value of [FT4] (40 pmol/L) and reducing to normal values, the [TSH] will normally respond after the compensation of the high level [FT4] effects. This also results in a shifted HP “curve 3” and therefore the associated shift in position of the set point.

In Figure 3, the set point shifts are demonstrated.

From Figure 3, it is easily appreciated that the shift of the set point (P1 and P3) compared to the original set point P2 is about ±4.5 pmol/L. The derivatives in all the set points are identical because of the unchanged exponential coefficient. From this example, we can conclude that the average value of the [FT4] values can be calculated to estimate the original set point when the hysteresis curves are the only data available.

## 3. Discussion

In general modern laboratory, biochemical assays for the determination of [FT4] and [TSH] values are not a subject for discussion related to the achievable measurement accuracy. However, the manner of reporting these laboratory values, together with the interpretation of laboratory results, can have a huge impact on the reconstruction and presentation of an individual HP characteristic. In this treatise, the implications of these measurement results are only related to a single individual in contrast to aggregated data of a large population where variations in these presentations are cancelled out by averaging procedures.

In the overview above, we presented a variety of uncertainty sources that can be encountered in [FT4]-[TSH] measurements. In order to reconstruct the HP characteristic of an individual in a reliable way, we have to ensure that the effects of extreme situations like long standing hypo- or hyperthyroid conditions are also taken into account. Furthermore, a standardized time of the day (e.g., early morning between 07.00 h and 10.00 h) for blood sampling for [FT4]-[TSH] measurements is necessary. A good knowledge of the laboratory measurement accuracy together with a more accurate representation of [FT4] values resulting in at least an extra decimal digit is often helpful. 

Over the years, the measurement technology and accuracy have improved tremendously. This means that measurements in the previous decade or earlier (e.g., years 2000–2003) are probably less accurate that the ones in 2012-2013. Only the measurement results of one and the same laboratory with the same calibration accuracy and methodological technique can be used in a set of measurements to determine the HP characteristic. It is important to realize that the approximation of [FT4] assay results is usually based on operationally standardized protocols adopted as a matter of specific laboratory practices for routine reporting. What is crucial to understand is the imprecision generated in terms of the deviations that can arise when such results are applied to automated mathematical modeling tools for the reconstruction of the HP characteristic.

There are of course other error sources like the monthly hormonal changes in women across the menstrual cycle which can influence the TBG effects on [T4] [13]. Besides the described uncertainties, there are many other factors, like gender, age, and possible hypothalamus-pituitary or thyroid defects, that can influence the measurement results.

When all dominant error sources, effects and interpretations are taken into account, we can use the laboratory assay values of [TSH] and [FT4] with the coefficient of variations to determine the impact on the accuracy of the predicted calculated HP curve.

## 4. Conclusion

In order to reconstruct a reliable HP function, the following dominant sources of deviations in TFT determination have to be taken in account including the recommended measurement regimens. We can distinguish some sources of uncertainties to be more dominant in their effects than others.

1. For general TFT measurement, the diurnal variations in TSH are dominant in the results for [TSH] depending strongly of the time on day that the value of [TSH] is established.
2. With the determination of [FT4] of persons using L-T4, for practical reasons, the blood sample is taken (preferably in the morning) before the daily dose of L-T4 has been administered.
3. For a consistent reconstruction of the HP characteristic, only TFT values from the same lab should be used ideally or as far as practicable.
4. Determination and reporting of [FT4] should preferentially have the accuracy to at least one decimal value (±0.05 pmol/L).

## Figures and Tables

**Table 1:** Illustration of absolute error [FT4] with different relative errors in [TSH] and different values of *φ*.

*φ*	*p* = 1%	*p* = 2%	*p* = 5%
	Δ[FT4] pmol/L	Δ[FT4] pmol/L	Δ[FT4] pmol/L
0.3	0.066	0.13	0.33
0.4	0.05	0.1	0.25
0.5	0.04	0.08	0.2
0.6	0.033	0.07	0.17
0.7	0.028	0.06	0.14
0.8	0.025	0.05	0.13

**Table 2:** 

[FT4] (*λ* = ±0.5)	*F*_*ε*_	[FT4] (*λ* = ±0.1)	*F*_*ε*_
15	±0.3	15	±0.05
10	±2.9	10	±0.45
5	±25	5	±4
